# Who’s “in the room where it happens”? A taxonomy and five-step methodology for identifying and characterizing policy actors

**DOI:** 10.1186/s43058-023-00492-6

**Published:** 2023-09-18

**Authors:** Gracelyn Cruden, Erika L. Crable, Rebecca Lengnick-Hall, Jonathan Purtle

**Affiliations:** 1 Chestnut Health System, Lighthouse Institute-Oregon Group, Eugene, OR 97401 USA; 2 Department of Psychiatry, University of California San Diego, San Diego, CA USA; 3 Brown School, Washington University in St. Louis, St. Louis, MO USA; 4 School of Global Public Health, New York University, New York City, NY USA

**Keywords:** Policy implementation, Policy actors, Policy implementation strategies, Policymakers, Policy

## Abstract

**Background:**

Engaging policy actors in research design and execution is critical to increasing the practical relevance and real-world impact of policy-focused dissemination and implementation science. Identifying and selecting which policy actors to engage, particularly actors involved in “Big P” public policies such as laws, is distinct from traditional engaged research methods. This current study aimed to develop a transparent, structured method for iteratively identifying policy actors involved in key policy decisions—such as adopting evidence-based interventions at systems-scale—and to guide implementation study sampling and engagement approaches. A flexible policy actor taxonomy was developed to supplement existing methods and help identify policy developers, disseminators, implementers, enforcers, and influencers.

**Methods:**

A five-step methodology for identifying policy actors to potentially engage in policy dissemination and implementation research was developed. Leveraging a recent federal policy as a case study—The Family First Prevention Services Act (FFPSA)—publicly available documentation (e.g., websites, reports) were searched, retrieved, and coded using content analysis to characterize the organizations and individual policy actors in the “room” during policy decisions.

**Results:**

The five steps are as follows: (1) clarify the policy implementation phase(s) of interest, (2) identify relevant proverbial or actual policymaking “rooms,” (3) identify and characterize organizations in the room, (4) identify and characterize policy actors in the “room,” and (5) quantify (e.g., count actors across groups), summarize, and compare “rooms” to develop or select engagement approaches aligned with the “room” and actors. The use and outcomes of each step are exemplified through the FFPSA case study.

**Conclusions:**

The pragmatic and transparent policy actor identification steps presented here can guide researchers’ methods for continuous sampling and successful policy actor engagement. Future work should explore the utility of the proposed methods for guiding selection and tailoring of engagement and implementation strategies (e.g., research-policy actor partnerships) to improve both “Big P” and “little p” (administrative guidelines, procedures) policymaking and implementation in global contexts.

**Supplementary Information:**

The online version contains supplementary material available at 10.1186/s43058-023-00492-6.

Contributions to the literature

- A methodology and policy actor taxonomy are introduced to overcome two challenges to advancing policy-focused dissemination and implementation science: identifying multi-level, cross-context spaces (or, policy "rooms") where policymaking and implementation planning occur, and identifying the specific policy actors involved.
- This five-step methodology can be used for identifying and characterizing policy actors involved across policy implementation phases and to inform participant sampling decisions.
- This methodology can help researchers better understand policy “rooms” that function as implementation contexts.
- The policy actor taxonomy and five-step methodology can aid researchers in developing effective policy actor engagement approaches tailored to policy actor types and “rooms.”

## Background

Health policy-focused implementation science and the related fields of health policy [1, 2] and public administration [3–5] share a goal of better understanding how policies can be rolled out to maximize population health benefits. Understanding how to optimize policy implementation requires knowledge of the multi-level contexts where policy is made and enforced, and of the actors within those contexts [6, 7]—all of which can be complicated and obscured by the focal policy’s scope and ever-evolving policy environment [8–10]. Policy “rooms” [11] are the places or contexts where implementation decisions occur. These include the formal forums for synchronous and asynchronous dialogue such as physical meeting rooms, video teleconferencing platforms, e-mail threads, and town hall meetings, and informal forums such as ad hoc meetings and hallway chats. More guidance on how to identify policy rooms and policy actors is needed to help researchers specify policy-relevant implementation contexts, develop research-policy partnerships, and accelerate the public health impact of evidence-informed polices [12].

Policies are often categorized as “little p” or “Big P” policies [1, 13, 14]. “Little p” policies (sometimes called “micro policies”) entail guidelines, procedures, or system-specific requirements (e.g., hospital human resource policies) that shape organizational and/or individual employees’ behavior. “Big P” policies (sometimes called “macro policies”) include mandates such as local municipality and state legislation, and federal statutes "that aim" to impact population health and the contexts in which individuals’ access and receive healthcare [6, 15–17], including through shaping which little p policies are possible. Big P policies have received relatively little attention in implementation science compared to little p policies [18, 19].

To inform efforts that maximize the population health impact of both policy types, this paper presents a taxonomy to aid implementation scientists in defining and understanding who is responsible for developing and implementing specific policies, as these actors are key study collaborators and/or participants. A five-step methodology is presented to identify policy actors in both policy types using a Big P case study and little p examples [20].

The proposed methodology aims to address two major hurdles to conducting policy implementation research: (1) meaningfully describing policymaking “rooms” where key policy decisions occur and (2) identifying the variety of policy actors responsible for policy design, adoption, preparation, implementation, and sustainment. Examples of important policy decisions include determining which problems to address, which intervention(s) to deploy and how, what money or resources to allocate, who is responsible for policy implementation and monitoring, and how to address unintended outcomes of policy implementation. Policy actors shape inner and outer implementation contexts through these decisions and by framing potential and observed impacts. They directly shape policy implementation by influencing how evidence is disseminated to other policy actors and the public (e.g., constituents), selecting who will be invited to and engage in subsequent policy implementation phases, and affecting the nature of implementation (e.g., sustained resource allocation, political will, intervention delivery) [6, 12, 20–24]. Identifying little p policy actors is often fairly straightforward, as the individuals work within discrete organizations and have readily identifiable roles (e.g., clinician, administrative assistant) [25–27]. Less obvious, however, are the myriad of actors and purveyors of knowledge or resources involved in Big P policy implementation [7]. To address these challenges, researchers need methodological guidance on how to identify the “room” and actors within [2, 12, 28, 29]. A five-step methodology is described below to support researchers in these efforts. But first, an expanded taxonomy of policy actor types that researchers can consider while applying the five-step identification process is presented.

### An expanded policy actor taxonomy

This policy actor taxonomy leverages existing literature and the authors’ policy-focused dissemination and implementation research experiences. The taxonomy can support identification of relevant policy actors across implementation phases and strategies.

A policymaker taxonomy presented by Bullock et al. (2019) also describes policy actor types (i.e., political actors, bureaucratic actors, special interests, experts, other) [6]. The current taxonomy differs in that it aims to specify policy actor types by their actions across policy pre-implementation (Exploration, Preparation), Implementation and Sustainment phases—develop, disseminate, implement, influence, enforce—rather than their daily job roles or characteristics (e.g., educate, provide clinical care). Conceptualizing policy actors by their actions (versus “political actor” or “expert” status, for example) might support both experienced policy researchers and researchers less familiar with policymaking and implementing processes or governance structures to start identifying individuals in policy networks relevant to their implementation support efforts. The current taxonomy’s breadth aims to accommodate the myriad of actors who shape how policies and related evidence are disseminated. Table 1 includes an overview of each policy actor type and how the current taxonomy aligns with Bullock et al. [6].

**Table 1:** Policy actor taxonomy

**Policy actor**	**Definition**	**Examples**
**Developer**	Individuals at the uppermost policy level who craft, revise, and pass policy that can be legally enforced or mandated through organizational levers.	- Political actors^a^ (politicians, elected federal, state, and local actors) legislators) - Service agency leaders - Legislative staffers and aids who assist in researching and drafting policy - Legal staff (e.g., lawyers, paralegals) who assist organizations or agencies in researching and drafting policy
**Disseminator**	Individuals responsible for communicating between policy developers and policy implementers about the opportunity or mandated need to adopt a policy, policy characteristics which components are mandated or adaptable, relevant timelines, and guidelines.	- Government staff in specific departments (i.e., not part of policy developer team) - Service agency staff - Academic research partnership knowledge brokers - Advocates and other special interest groups - Media^a^
**Implementer**	Any individual with responsibility for decisions during implementation planning, active implementation, or policy sustainment. Might overlap with policy developers.	- Leadership, middle managers, front-line workers - Special interests^a^ (implementing agencies, street-level bureaucrats)
**Influencer**	Individuals or organizations who disseminate evidence (scientific, practice-based, anecdotal) to influence what evidence is trusted and how evidence is used in policy and policy decision-making.	- Lobbyists - Advocates and other special interest groups - Voters who participate in public testimony or public comment periods to shape policy - Special interests^a^ (donors/foundations, government corporations, unions) - Experts^a^ (scientists/researchers, patients or persons with lived experience and families/caregiver, field or practice leaders/champions, innovation/developers and disseminators (purveyors), intermediaries and technical assistance providers) - Media^a^
**Enforcer**	Individuals who are tasked with monitoring policy compliance. Might overlap with policy disseminators at one level, and with people responsible for compliance within the implementing level (another level).	- Government oversight committee - Healthcare, other insurers^a^ - Executive departments^a^ - Boards and agencies of government^a^ - Self-governing regulatory agencies - Judicial system^a^

^a^Bullock et al. 2021 taxonomy

Policy actors include any individual who might be responsible for decision-making regarding the design and implementation of Big P or little p policies. Policy actors’ roles, responsibilities, and points of influence can vary as the policy is implemented across multi-level policy contexts [6] and implementation phases. This is especially true in small agencies where boundary spanning is common (i.e., serving as both a regional administrative director and clinical supervisor in a human services agency) [30]. The typology includes five categories of policy actors (developers, disseminators, implementers, influencers, and enforcers) across policy types (Big P or little p), contexts (inner, outer), and the four, non-linear phases of implementation outlined by the EPIS framework (Exploration, Preparation, Implementation, Sustainment) [12, 31].

*Policy developers* play a critical role in determining which problems are addressed and shaping the vision or intention of a policy to address these problems. They help determine which outcomes matter, which population(s) to target [32], how change should be achieved, and which resources to allocate. They delineate which decisions they will continue to make and which will be made by other types of policy actors [6]. They can be elected or operate in an administrative role [33]. Policy developers often play roles in Exploration and Preparation. They may or may not be involved in Implementation and Sustainment phases. Conversely, policy disseminators, implementers, and enforcers—described below—play instrumental roles in those latter phases.

*Policy disseminators* decide who should be notified about the policy to ensure policy institutionalization and how relevant information should be spread, particularly during Preparation. They hold a unique position of power critical to shaping the policy transfer process. They include federal/state agency staff tasked with integrating new policies into their agency’s operations and communicating information about the policy with relevant actors such as healthcare providers and insurers. Policy disseminators usually originate from the policy developing institution but might also reside in third-party organizations with relevant subject-matter expertise or interest [34].

*Policy implementers* can include elected or appointed federal and state officials, healthcare providers and insurers complying with Big P policy, or individuals responsible for implementing little p policies passed down from organizational leadership (i.e., policy developers) [33, 35]. This definition aligns with Leeman et al.’s definition of “*delivery system actors*” who adopt and integrate evidence-based practices and policies (EBPs) into their practice settings [34] but adds a focus on these actors’ roles in policy implementation and their decision purview. Policy implementers might have day-to-day decision-making authority in their organization, service setting, or other unit of jurisdiction, or have unique authority given a specific policy.

*Policy influencers* impact which evidence is used and how at any phase of policy implementation, although they are particularly influential during policy development (Exploration), dissemination, and Sustainment [36–38]. Influencers include both formal (e.g., organized advocacy groups) and informal actors (e.g., colleagues, local media) [6, 39, 40] within a policy network. Mixed-methods and audience segmentation [41, 42], in particular, could be useful to further specify policy influencers’ formal and informal roles, the type of information they share with other policy actors, and their degree of persuasion.

*Policy enforcers* communicate with policy disseminators and implementers about implementation success during Implementation and Sustainment. They can operate within a local organization (e.g., hospital, health insurer) for internal monitoring or in external, non-government watchdog organizations. However, most are employed by government agencies [43]. Policy enforcers and disseminators might also take on the role of *support system actors*—actors within or outside of a delivery system that provide support, such as technical assistance or facilitation, to increase EBP adoption and implementation quality [34].

### Challenges to identifying policy actors and “rooms”

Despite multiple calls for better specification of policy actors and their evolving roles in implementation efforts [6, 12, 34, 44, 45], there is a lack of methodological guidance about how to actually use these types of conceptual taxonomies in research design decisions. Furthermore, identifying policy actors is not straightforward, as explained in the three challenges outlined below.

#### The diversity of actors who impact policy implementation decisions over time

Policy implementation requires continuous decision-making by individuals who operate in both formal (e.g., organizational implementation lead, consultants) and informal (e.g., knowledgeable individual whose input is unofficially invited but considered) roles. This plethora of actors and role ambiguity makes it challenging to identify who is responsible for and wields influence over policy implementation at a given timepoint [7, 12, 46]. For instance, compliance requirements, such as which EBPs are eligible for funding, require a “room” of actors from payor organizations, government, and regulatory entities (i.e., policy enforcers) [47]. Once a policy is created, it must be transferred and adopted by a provider organization. These decision makers and front-line staff then take on local policy implementation roles. Additionally, some actors, such as lobbyists, influence initial policy decisions through their formal roles in the outer policy context [6, 48], but may lack necessary sway to achieve policy transfer to the inner context and not play a critical role in ongoing policy implementation processes or policy outcomes [1, 39, 40, 49, 50].

#### Policy actors’ professional identities are inconsistent and sometimes masked

There is substantial heterogeneity in the roles and titles that government agencies and non-governmental organizations give to policy actors. Individuals at higher levels within organizational hierarchies typically have final decision-making authority. Yet, in the absence of an organizational chart and written role descriptions, it can be challenging to determine whether a “Director” or “Assistant Director” has the uppermost decision-making authority, whether these position titles are equivalent across agencies (e.g., social services, public health) or settings (e.g., government agency, non-government organization), and whether they have the same level of decision-making authority across different policy issues (e.g., youth vs. adult services). Even when organizational hierarchies and decision-making processes are decipherable (by reviewing public meeting minutes, recordings, and reports), much of policy decision-making occurs behind the scenes [46, 51]. Furthermore, decisions critical to policy implementation are often made by frontline workers (i.e., policy implementers) or, “street-level bureaucrats” [52], who rarely appear in organizational charts or whose title in such charts does not fully convey their policy implementation responsibilities [6]. For example, while the Director of Child Welfare may have authority to decide which EBPs will be adopted [53, 54], other actors, such as case workers’ supervisors, might influence their decision and how it is implemented.

#### Opportunities for identifying policy actors are often time-limited

Policy implementation often occurs with mandated commencement and/or expiration timelines, and sometimes relies on “policy windows”—opportunities for a clearly defined problem to converge with both a proposed policy solution and political support for change [7]. Policy formation and implementation is affected by factors such as political will, resource availability, public support, and competing demands that shape the policy implementation window. These forces increase the need for practical approaches to rapidly identify policy actors so that implementation strategies can be developed to open the “policy window” and nimbly respond should the window open or shift.

## Methods

**Fig. 1:**
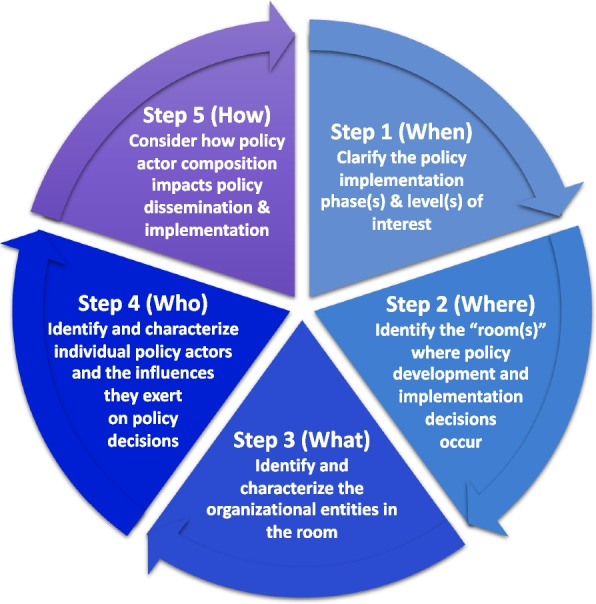
Five-step methodology for identifying the policy “room” and actors within it

A five-step methodology was developed to address these challenges to provide concrete steps for identifying relevant policy “rooms” used during specific policy implementation phases and the actors within those “rooms” (Fig. 1). The steps were developed to guide sampling decisions for a study (i.e., the case study presented below) that aimed to support state-level decision-making during implementation of an optional federal policy. The policy’s extended roll-out warranted a reproducible method for mapping and documenting the “room” over time and across sites.

### Case study: Family First Prevention Services Act of 2018 (FFPSA)

FFPSA is a federal policy to prevent child maltreatment, reduce out-of-home placements for children, and increase permanency should children be removed from the home [55]. FFPSA makes federal funding available for states to implement EBPs targeting parenting practices, family functioning, parent mental health and substance use, and child behavior. To receive federal funds, states, federally recognized tribes, or US territories had to submit a 5-year prevention plan (“State Plan”) describing their rationale for implementing specific EBPs or other interventions. FFPSA specifies that the State Plan detail “a description of the consultation that the State agencies responsible for administering the State plans…engages in with other State agencies responsible for administering health programs, including mental health and substance abuse prevention and treatment services, and with other public and private agencies with experience in administering child and family services, including community-based organizations” [55]. Thus, FFPSA required states to work with multiple policy actors, and it was not straightforward to predict or immediately identify who was in the “room.” As of December 2022, 36 states and the District of Columbia submitted a plan [56].

### Identifying sites and gathering materials for document review

The pilot study aimed to support state-level decision-making around EBP adoption in response to FFPSA, so researchers needed to identify which states (a) were in Exploration and still in the process of deciding or (b) had decided which EBPs to adopt (i.e., advanced to Preparation phase).

Using a snowballing approach, state-specific, publicly available resources (e.g., websites, briefings, presentations, State Plans) were gathered using a web-based search (e.g., “[state name] FFPSA”) and a targeted search on the federal policy enforcer’s website (Administration of Children, Youth, and Families) to identify organizations and individuals involved in Exploration. State Plans were gathered first, as these public-facing documents were mandated and partially standardized through FFPSA, increasing harmonizability across sites. The state-level institution leading a state’s FFPSA implementation, such as a department of health and human services (hereafter, “lead organization”) was identified while searching for plans because these institutions often disseminated and/or were named in the State Plan. Lead organizations’ websites and those of related state agencies were combed (e.g., department of social services, governor’s office), as were websites maintained by non-profit organizations monitoring FFPSA implementation (e.g., Casey Family Programs).

Organizations and policy actors were identified through State Plans and a web-based search using policy and state-specific terms (e.g., “FFPSA workgroup [state name]”). Some states listed only the involved organizations or specific policy actors’ names, while others offered both the policy actors’ and organization names. For states with specific organizations or policy actors listed, the names were entered into a web-based search to identify or verify organizations where policy actors were employed or volunteered, policy actor’s position within the organization, policy actor’s contact information, and the organization’s mission statement or other documentation that might be useful for characterizing organizations and actors (e.g., organizational charts). Notable sources included media reports, organizational newsletters, organization websites, personally managed websites, independent news, professional networking sites such as LinkedIn, and FFPSA-related databases curated by research and non-profit organizations [57].

When there was conflicting information across sources, the most recent source was used. When no dates were available, the source with the most complete information was used. Policy actors’ personal websites and professional social media (i.e., LinkedIn) were particularly helpful for clarifying actor’s roles when they were in the “room,” as it was not uncommon for individuals to obtain new positions or change organizations since being in the “room” and the most accessible information was often on their new organization’s website.

### Code development and refinement

Two coders trained in policy research, implementation science, and qualitative methods (GC, RLH) were responsible for data analysis. The initial coder (GC) developed emergent codes for organization and policy actor types using a subset of data units (*n* = 10 states). Coders met to adjust codes for generalizability and parsimony. A team member (JP) provided feedback on the revised codebook that was then applied by the initial coder to 20% of the data units. The two coders again met to revise codes and code definitions before the initial coder applied the revised codebook (Additional file 1) to all data and made detailed memos in a web-based spreadsheet that detailed code application justification. The initial coder also recorded if information was missing, such as when organizational charts and mission statements were not available or role purview was ambiguous.

The second coder (RLH) completed a line-by-line review of code applications for all data units. They then independently generated memos that reflected on new codes, themes, and coding challenges. Codes emerged for both organization and role types. New codes were deemed necessary if the additional detail they offered would characterize the “room’s” homogeneity or diversity (and thus identify potentially missing actors/voices, or jurisdictions), identify potential power dynamics, or further characterize the history of decision-making to better understand what challenges or opportunities might need to be navigated in a policy implementation study. Finally, the coders met to resolve coding discrepancies.

Mission statements informed organization type codes. Codes ultimately distinguished organizations by contextual level (e.g., state, community) and purview (e.g., community-based services, advocacy, lived experience). Lower levels of hierarchical service systems, such as county child welfare directors operating underneath state-level departments, were counted as unique organizations because they have separate decision-making processes and authority over policy implementation.

Coding policy actor roles required understanding each organization’s personnel chart and mission, not just based on the organizational role title alone. For example, “deputy director” in one state might be considered “executive leadership”—the highest leadership level—while a deputy director might be coded as “leadership”—a lower-level position—in another state. Role codes were distinguished by leadership level, proximity to frontline service provision, professional expertise, and lived experience. Role categories were mutually exclusive for the illustrative policy (FFPSA) but, outside of the target effort, these roles might overlap due to real-world professional responsibilities. For the case study, it was more important to broadly characterize individuals’ roles rather than capture extensive detail. This decision might vary for other policy implementation studies. In addition to their organization-specific role, actors’ proximity to policy decisions also shaped role codes. For example, programmatic roles were separated into “administrative” and “direct client care” because those providing services were not expected to have decision-making authority over EBP adoption.

### Content analysis

To facilitate broad comparisons of the “rooms,” content analysis—a method for systematically characterizing themes in textual data by sorting text into well-defined categories based on coding rules [58–60]—was employed. Each code and code category (i.e., individuals named, organizations listed, no organizations or individuals listed) was assigned an indicator variable (1, 0). Next, descriptive statistics (e.g., counts) were created in the spreadsheet where codes were assigned. Memos were again created by the two coders at this stage to reflect on who was in the “room” and the policy implementation progress.

## Results

### Overview of five-step methodology for identifying policy actors

Step 1 clarifies the policy implementation phase(s) and level(s) of interest. Step 2 identifies the proverbial or actual “room” where policymaking decisions and actions of interest occur. Next, the process identifies and characterizes the organizations or entities (step 3) and individual policy actors (step 4) in the “room.” The process concludes with quantitatively and qualitatively comparing the focal policymaking “room(s)” with other policy-relevant contexts and “rooms” (step 5). The goal of this comparison is to understand which voices might be missing, identify potential power dynamics between key parties (i.e., policy actors and policy recipients), and begin conceptualizing how sampling and engagement approaches—such as mutually beneficial messaging for study recruitment and dissemination—might be tailored to each “room” [61–63]. Table 2 provides an overview of each step and illustrative insights from the case study.

**Table 2:** Five-step methodology with FFPSA illustrative case study

**Step**	**Purpose**	**Illustrative case study**
**1**	Primary: Determine which policy implementation phase (e.g., Exploration, Preparation, Implementation, Sustainment) is of interest and at which level (e.g., state, federal).	**Case study definition**: The current project aimed to support EBP adoption decisions at the state level in response to a federal policy, FFPSA.
	Secondary: identify *sites* by their likely policy implementation stage, including whether potential sites will be adopting a policy.	**Data sources/search techniques: **Sites were identified by consulting a federal agency website (Children’s Bureau, an Office of the Administration for Children and Families) that reported whether states had submitted, received approval, or not yet submitted plans for federal approval to implement FFPSA. (Accessed 29 August 2022). Considering that there might be a lag in reporting on this federal website and that some states might be actively preparing their plans, state-specific websites were also searched by entering the policy and state name in a web search, or by searching the official website for the state agency likely to be responsible for implementing the policy (e.g., department of social services).
**2**	Identify who is involved in policy decisions during the focal implementation phase, as these individuals can become key actors or targets of implementation strategies.	**Case study definition**: The group of individuals involved in shaping the State Plan comprised “the room.” States were categorized as having enough information to identify the room and included in step 3, having insufficient information, or as being non-adopters of FFPSA.
		**Data sources/search techniques: **Manually searched the official website for the state agency likely to be responsible for implementing the policy (e.g., department of social services) and reviewed any available documentation such as the submitted or approved State Plan and meeting minutes or presentation documents (e.g., Power Point).
		**Results: **States were considered as being in *Exploration* if they had not submitted a plan but sufficient information was available to determine the state’s intention to submit (*n* = 1) or if they had a submitted (*n* = 15) or federally approved plan (*n* = 21). Some states had a singular “room” where all FFPSA decisions were made, while others had multiple “sub-rooms” (i.e., workgroups). For example, some states had workgroups focused on specific implementation activities such as identifying which evidence-based practices to adopt (e.g., “Prevention Subcommittee” with In-home parenting, Mental Health, and Substance Use Disorder Workgroups-Ohio; “Services Continuum”-Colorado) or “sub-rooms” comprised specific actors (e.g., “FFPSA Leadership Team, FFPSA Stakeholder Workgroups”-Maine; “Leadership Advisory Team”-North Carolina).
**3**	Characterize the organizations in the room to help describe actors’ roles and perspectives.	**Results:** Ten states detailed organizations involved in state prevention planning for FFPSA, with a range of 1–41 specified organizations per state. Two additional states broadly referenced organizations or types of organizations that were involved in decision-making but did not specify all organization names (for example, simply provided a descriptor such as “community providers, judiciary, other state agencies, private sector businesses”) or individuals associated with decision-making. Organization types included state and local government, non-governmental service agencies, advocacy or lived-experience, and research-oriented. Individuals with lived experience represented their own “organization” type and thus were counted as unique organizations. The state’s child welfare, social service, or other human services agency responsible for that state’s child welfare cases was consistently present, whereas leadership from smaller jurisdictions from the relevant agency were inconsistently or incompletely present (e.g., some county or regional directors but not all).
		**Data sources/search/analysis techniques: **Conducted web-searches for organization-specific websites to extract their mission statements or, in the absence of a mission statement, review what services are provided (often under a site tab such as “About us” or “What we do”). When available, mission statements were directly pasted into the codebook to facilitate reflection on why specific codes were created and/or applied. Often gathered organization charts at this point.
		**Potential analyses and alternative considerations: **A range of qualitative methods could be applied, such as rapid qualitative analysis and content analysis, as well as more descriptive analyses (e.g., matrices with binary indicators by stated role type).
**4**	Identify the extent to which policy actors from step 3 might influence decision-making in subsequent policy implementation phases by characterizing roles, responsibilities, and actions.	**Results:** Eight states listed specific individuals involved in FFPSA planning and/or active implementation, ranging from 2 to 87 individuals per state. Individuals were coded into 13 categories distinguished by leadership level through their organizational role and their organizational affiliation. Roles and organizations could not be identified for 15 individuals. The majority of individuals (67%) were in leadership roles at their institution. These policy actors, in particular, could be important partners in the policy *implementation* phase. A relatively small group (4%) of individuals brought a lived experience perspective. All but four of these individuals seemed to be invited to the room through their organization, such as employment or volunteering through a community advocacy organization. These individuals were from the same state. In reality, input from those with lived experience might have been much higher, as most states described hosting public listening sessions such as town halls. Because these individuals were not listed as part of the room and did not have decision-making authority regarding policy implementation, they were excluded from frequency counts (step 5).
		**Data sources/search techniques: **Similar to step 3, organization-specific websites were searched, as were professional networking sites such as LinkedIn. Individual roles and/or organizational affiliations were sometimes most easily identified through reports released by their respective agency in which the report was prepared or signed by the individual. Local news articles or organization newsletters also provided important details, particularly when an individual was no longer in the role that they assumed when the policy room was originally formed.
		**Potential analyses and alternative considerations: **Crable et al. (2022) [12] provide further recommendations for specifying and reporting how policy actors’ roles, responsibilities, and actions might vary throughout policy implementation. Presseau et al. (2019) [45] provide a framework for specifying actions. Systems science methods such as agent-based modeling and causal loop diagramming could aid hypothesis generation and simulation-based testing of these hypotheses, such as potential engagement and implementation or dissemination strategy specification choice impacts on implementation and policy outcomes.
**5**	Identify which perspectives or interests might be more saturated than others.	**Results**: There was extensive heterogeneity in the size, composition, and structural processes of each state’s room. Some states including several branches of state government, representing a potentially broader, more comprehensive implementation compare to states that only included the lead organization.
		**Data sources/search techniques: **This stage did not require new data sources. However, it did often require revisiting sources from other stages to interpret organization and professional roles within the context of one another.
		**Potential analyses and alternative considerations: **Descriptive statistics (e.g., tallies), rapid qualitative analysis, landscape analysis, audience segmentation, social network analysis.

### Step 1

The primary purpose of this step is to clarify which policy implementation phase (e.g., Exploration, Preparation, Implementation, Sustainment) is of interest and relevant level(s) of implementation (e.g., state, federal). The same types of policy actors (e.g., developer, disseminator) might concurrently exist across levels for a given policy, but with different purviews. For example, while a Big P policy is created by policy developers at a higher level (e.g., federal), communication and active implementation responsibilities are often handled by policy disseminators and implementers at a lower level (e.g., state). There is rarely a single “room” for each policy. Furthermore, Big P policies might not require that all eligible levels or sites (e.g., states, service systems, organizations) adopt the policy. Thus, a secondary purpose of this step is to identify sites by their likely policy implementation stage and policy adoption status.

### Step 2

This step aims to identify the “room(s)” within each site, including who is involved in policy decisions during the focal implementation phase(s) [44]. “Room(s)” of interest (step 2) might vary based on the prioritized phase and level(s) (step 1), as the policy actors might vary by implementation phase or the same policy actors might assume different roles across implementation phases [12], thereby generating new “room” compositions. When the “room” is a physical, easily discernible place such as the legislative floor where a policy is debated, a town hall meeting, or a board meeting, such “rooms” typically serve as the “room” for multiple policies or implementation phases. In contrast, some “rooms” are formed only for a given policy (e.g., temporary, cross-sector workgroups) or phase and might exist in fluid or non-physical locations (e.g., phone conversation, email exchange, or virtual meeting space). These shifting “rooms” are only identified by the congregation of policy actors. While identifying the “room,” it is likely that organizations and/or policy actors in the “room” will also be identified. These data should be captured for steps 3 and 4.

This step can require consulting multiple, diverse resources. For example, some public policy procedures, such as legislative sessions and town hall meetings, are live-streamed or recorded for asynchronous access on organization-specific websites or general domains such as YouTube. These resources can illuminate who was not only in the “room,” but who engaged in conversation and decision-making. Freedom of Information Act requests can provide access to meeting documents and policy materials that are not readily available, though such requests can often take months or years to be fulfilled. Once there is relative confidence that the “room” has been identified, it is time to advance to step 3.

### Step 3

This step aims to characterize organizations in the “room” and to describe actors’ roles. For these purposes, “organizations” can represent a variety of sampling units including for-profit or non-profit organizations, service systems, or informal organizations and perspectives. Discrete organization categories were derived during the case study, described below and in Additional file 1. The categories were created to be generalizable across policy implementation studies. However, specificity might vary by policy or setting. Guiding questions about optimal code specificity include: Does going more fine-grained help identify which organizations and actors have the most or least power or jurisdiction? What organizations/entities might be missing?

### Step 4

The purpose of this step is to identify the extent to which policy actors from step 3 might influence decision-making in subsequent policy implementation phases. This is accomplished by identifying each actor’s professional roles and responsibilities, then categorizing these roles by authority level or purview (e.g., state, county, community), responsibility (e.g., leadership, middle manager, administration), and perspective (e.g., health, judicial, lived experience). Formal organizational roles and responsibilities do not always encompass the scope of actors’ professional activities and therefore influence. Capturing specific activities for which actors are involved (e.g., giving presentations, writing reports) can be illuminating. Behavioral frameworks can help specify policy actors’ behaviors if actions connote different roles and responsibilities than static information such as a professional title [45, 64]. The policy actor taxonomy proposed here and/or the taxonomy proposed by Bullock et al. [6] can help characterize roles and responsibilities. For example, the policy implementer might be further specified by sub-types within Bullock et al.’s taxonomy such as the “implementing agency” [6]. Additional file 1 demonstrates how the proposed coding structure could be overlayed with the Bullock et al. taxonomy. Systems science methods such as causal loop diagramming [65], agent-based modeling [66], and social network analysis [67] can capture interdependencies in actors’ activities that cause other activities to be delayed, increase, or decrease. For example, increased advocacy by an *influencer* could increase a *developers*’ motivation to draft a bill. This positive outcome could in turn encourage *influencers* to increase their advocacy for other bills or maintain engagement in subsequent policy implementation phases for the original policy.

### Step 5

This step aims to identify which perspectives or interests might be more represented than others within a particular “room” during a particular implementation phase, as sites can be in multiple phases simultaneously or re-visit phases [12, 68]. This step can help researchers and their implementation partners reflect on whether the room is missing actors whose perspectives are critical to holistically understanding the policy or problem to be impacted, achieving implementation and intended policy outcomes, and/or anticipating unintended consequences. Sampling and engagement approaches can be directly informed by the heterogeneity (or lack thereof) of policy actors in the “room” as indicated by the policy actor taxonomy (i.e., their implementation role) or their interests and expertise (e.g., health, education, child welfare, lived experience). For example, if a particular service system will be responsible for day-to-day policy implementation and is not in the room during Preparation, a study focused on supporting Implementation might want to ensure that sampling strategies extend beyond the Preparation “room” to include these important actors [69].

## Discussion

An enhanced taxonomy of policy actors was introduced to help implementation scientists characterize the roles that specific policy actors play throughout policy development and implementation. A five-step methodology for identifying specific actors and the “rooms” in which they congregate was also introduced. This methodology was designed for researchers who want to engage these actors in implementation research and characterize policy implementation contexts. Addressing calls to specify the contexts in which policy will be implemented [12], the methodology offers standardized (e.g., policy and context agnostic) approaches for characterizing one aspect of the policy context—the “room”—and actors that shape policy development and implementation. By carefully characterizing the room, implementation scientists can be better prepared to understand policy implementation barriers and facilitators, identify which actors are key for ensuring quality policy implementation, and successfully approach these actors as research partners and participants.

### Contributions of the five-step methodology

Researchers can employ this methodology for any policy implementation study and any phase. The steps are intentionally designed to handle the inherent fluidity, dynamism, and non-linearity of this type of research. Each step can be revisited as new data sources are available (e.g., town hall meetings, public testimony), but such recursiveness does not require repeating all steps in order. Knowledge gained can guide study design, sampling, and engagement approaches that are feasible and appropriate given the identified actors in each “room.” This focus on guiding sampling and identification of both *who* and *what* activities can be supported through implementation strategies distinguishes it from existing behavior identification frameworks, such as the Actor, Action, Context, Target, Time (AACTT) framework [45]. AACTT is primarily operationalized in contexts for which the actors are known (i.e., nurses and administrators in a health service organization). The current methodology offers steps to identify specific policy actor roles and activities while accounting for ambiguity in who might be involved in policy implementation.

This methodology primarily relies on publicly available information. Therefore, it is widely accessible and can be rapidly employed to respond to evolving policy windows and implementation timelines. Rapid identification could be critical to intervening upon the policy implementation process [8]. As policy actor-research partnerships are developed, partners can provide additional data for coding and comparison in steps 3–5 [70] and validate or modify characterizations of the “rooms,” other actors, and policy implementation processes.

This methodology is useful for guiding study sampling decisions, but knowledge generated through these five steps can support researchers to operationalize policy-level dissemination or implementation strategies. Researchers need to be clear about which policy actors are or will be involved/targeted by an implementation strategy in order to specify what cognitive processes, behaviors, or other mechanisms specific to those actors will be targeted for modification [44]. This is acknowledged in existing implementation science frameworks, such as AACTT [45], which specifies behaviors to be modified by implementation strategies. However, actions specified through the current methodology may or may not be targets for modification. Policy researchers have repeatedly documented how different policy actors’ knowledge [71], values [72, 73], beliefs [41, 74], and decision-making authority [24] can drastically influence policy outcomes. For example, research suggests that US policy actors’ partisanship strongly influenced state-level policy responses to the opioid epidemic [75]. Policy implementation strategies should be tailored to policy actors’ values, expertise, and evidence-use behaviors to effectively target mechanisms in their decision-making processes [71, 76–78].

### Suggestions for using the five-step methodology

#### Report consistently

To enhance the harmonization of applications, it is recommended to report this methodology with a level of detail consistent with qualitative reporting guidelines (e.g., COREQ [79], SRQR [80]—Additional file 2). Additional file 3 includes prompts for additional details. While the proposed high-level codes can be consistent across applications (e.g., state government, tribal services, lived experience), the exact codes can vary by policy and context.

#### Maintain detailed documentation

Organization and role codes must be carefully defined and applied. For example, if researchers find it important to distinguish between “executive leadership” and “leadership,” they should record the criteria and rationale for this distinction.

Qualitative memos are critical for informing implementation study design and engagement approaches. In the illustrative example, memos focused on how transparent a state’s decision-making process seemed, how recently active the “rooms” were, and whether there were existing research partnerships and opportunities for additional research-practice partnerships. Alternative observations might include: documenting when a policy actor’s role might be activated during policy implementation (i.e., when their decision-making authority might be most influential) and actor-level details such as their prior stances on policy issues, priority areas [81], and collaborators or frequent opponents.

Due to the evolving nature of policy making and implementation, data can quickly change, making record-keeping (and the ability to retrace previous decision-making) especially important. Researchers should capture source locations (e.g., website links) and archive documentation (e.g., screenshot organizational charts, download PDFs).

#### Allocate ample time

Researchers should budget ample time for applying this methodology and for developing deeper contextual knowledge of the policy environment. Conducting the case study searches took approximately 40 h, while coding took another 25 h (including co-coding and resolution). These estimates do not include time spent reading each State Plan and learning about the policy to anticipate and understand the potential areas of divergence in policy implementation across contexts.

The scope and time required will likely increase as the number of policy implementation levels increases. For example, in addition to jurisdiction-based nesting (e.g., state, county), another type of nesting occurs when a policy requires implementation of subsequent policies or numerous EBPs. FFPSA is an example of a multi-nested implementation effort. States implement a federal policy by implementing multiple EBPs. Furthermore, multiple service systems (e.g., state-level child welfare, education) or organizations within a singular service system (e.g., regional child welfare entities) might be involved. As the number of actors increases, so does the number of simultaneous implementation processes (e.g., coordinating shared resources for the same target population) and the number of “rooms.”

### Limitations

This methodology can aid researchers in identifying many, but likely not all, policy actors in the “room.” Given the potential ambiguity and incompleteness of available data to inform coding and decisions across the steps, it is strongly recommended that at least two coders review extracted data. Coders should strive to achieve a shared understanding of the political contexts and actors shaping policy implementation decisions to inform policy actor engagement and implementation strategies. Varying specificity is expected when this methodology is applied to new policy implementation contexts. To promote greater specification of actor’s actions and roles, future research can verify how the current categories are associated with real-world observations of policy actors’ influence on policy implementation and how actor descriptions overlap with roles and characteristics specified in related taxonomies [6].

These steps rely primarily upon public information that can become quickly outdated. Every effort was made to use alternative sources that might be more up-to-date, such as personally managed social media accounts and news websites. The flexibility of the proposed methodology allows researchers to iteratively apply the five steps as the policy is rolled out and as new data are available.

### Potential methodology adaptations and considerations

All five steps might not need to be completed or conducted in the proposed order before engaging policy actors or specifying the potential policy implementation project scope. Although information in each step informs the next, rapidly changing policy windows and funding opportunities might necessitate abbreviating methodological considerations in each step or skipping some steps entirely. Understanding which steps are most appropriate given study goals, the focal policy implementation phase(s), and study resources could foster greater efficiency and rigor in applying these steps. After identifying the focal policy implementation phase (step 1) and at least some characteristics of the potential policy actor sample (i.e., the categories by which you might code organizations and actors in steps 3 and 4), it might be more efficient to reach out to intermediaries (e.g., *policy influencers*) or readily identifiable policy actors to directly inquire about other relevant actors (similar to snowball sampling). These point-of-contact actors include those who present at public forums, write reports, and serve in public-facing roles (e.g., program managers). Media relations contacts are not policy implementers or developers but are often identifiable and can be useful for initial outreach.

Future research should explore whether variations in how the steps are applied impacts the efficiency and accuracy of identifying who is in the “room” and their actions. For example, accuracy could be assessed by triangulating researchers’ codes and code assignment (steps 3 and 4) with actors’ self-reported influence on policy implementation. Accuracy might also be assessed by calculating the percentage of actors who were not identified through the proposed methodology after confirming the “room” with policy actors. Relatedly, while the case study demonstrated the methodology’s utility during study design while a policy was in Preparation or early Implementation, future research can compare the feasibility and utility of applying the proposed steps to support mid-Implementation or Sustainment activities, or for retrospective policy evaluations.

Both the taxonomy and proposed steps might be refined with additional data structures (e.g., social network ties) or types (e.g., stances on prior policies or issues). Such information could help identify how actors’ actions overlap and vary by policy and policy implementation phase. Social network analyses and individuals’ issue positions, in particular, could illuminate which policies might be supported or face barriers, which actors are instrumental across policies and thus might be leveraged to deliver or be targeted by dissemination and implementation strategies [33, 41, 42], and which perspectives might be under-represented.

### Conclusion

Knowing which policy actors are involved, their scope of influence, and when influence is exerted in policy implementation processes is critical for designing effective policy implementation studies. This article provides researchers with a policy actor taxonomy—developers, disseminators, implementers, influencers, and enforcers—that transcends professional roles while accounting for policy implementation phases. A methodology is provided to assist researchers in identifying and characterizing these policy actors in diverse policy implementation efforts.

### Supplementary Information

**Additional file 1.** Codes Applied in Steps 3, 4 for FFPSA Case Study.

**Additional file 2.** Standards for Reporting Qualitative Research (SRQR) Checklist.

**Additional file 3.** Suggested Reporting When Applying Five-Step Methodology for Identifying the Policy “Room” and Actors Within It.

## Data Availability

The datasets used and/or analyzed during the current study are available from the corresponding author on reasonable request.

## Abbreviations

EBP: Evidence-based programs and policies
EPIS: Exploration, Preparation, Implementation, Sustainment Framework
FFPSA: Family First Prevention Services Act of 2018
US: United States
