# Supplementary material for: Who’s “in the room where it happens”? A taxonomy and five-step methodology for identifying and characterizing policy actors
Source: Implement Sci Commun. 2023 Sep 18;4:113. doi: 10.1186/s43058-023-00492-6 (PMC10506261; doi:10.1186/s43058-023-00492-6)
Supplement: Supplementary file 1 — Additional file 1. Codes Applied in Steps 3, 4 for FFPSA Case Study. [file 43058_2023_492_MOESM1_ESM.pdf]

Additional File 1: Codes Applied in Steps 3, 4 for FFPSA Case Study

Initial Codes	Iterated Code (if applicable)	Description	Examples from FFPSA Case Study
**Organizations (Step 3)**			
**National-level**			
National advocacy	—	Has organizational mission statement to advocate for a particular population or issue. Typically, a non-profit.	Youth Villages; Mental Health America; National Youth Advocate Program
**State-level**			
Bureaucratic/political*			
State legislature	+	State-level government body and supporting entities such as administration	State Representative; Office of the Governor; Senator's Office
	State government- social services	State agency or department responsible for delivering benefit programs such as food assistance (e.g., State Nutrition Assistance Program-SNAP), unemployment, and individual or family social services unrelated to child protective services activities	Department of Human Services
State government- child welfare	—	State agency or department responsible for child protective services and making determinations of child safety and custody	Department of Human Services; Department of Job and Family Services; Department of Child Welfare; Office of Children, Youth, and Families; Cabinet for Health and Family Services
State government- judicial	+	State agency or department responsible for the legal system (e.g., courts)	[state] Supreme Court; Court Improvement Project
	State government- justice/corrections	State agency or department responsible for administration of justice system (e.g., prison, law enforcement)	Department of Justice; Division of Juvenile Services
State government- Medicaid/health	+	State agency or department responsible for public health, Medicaid, and other health services	[state] Health Authority; Department of Medicaid; Department of Human Services; Department of Public Health and Environment; Health Care and Public Financing
	State government- disabilities	Often part of a state health authority but unique department responsible for the needs of disabled individuals. Often nested within the state health authority but not always	Department of Disabilities; Office of Developmental Disabilities and Support Services
	State government- mental health	State agency or department responsible for public mental health and/or behavioral health. May or may not include substance use	Department of Mental Health and Addiction Services; Department of Behavioral Health;
State government- education	—	State agency or department responsible for public education system	Department of Education

State government- other	—	Any state department or agency not reflected in more specific codes or that crosses several codes	[state] Youth Services; Policy and Communications
Independent oversight agency within state government	—	A government organization that is independent of those providing services; mission is to provide oversight, auditing, and/or evaluation	Foster Care Review Office
**Regional/Local-level** (e.g., county, regional)			
Bureaucratic/political*			
Regional government- child welfare	—	Regional government agency tasked with child welfare and family services at any level other than state, including regional and county	[county] Child Services Agency
Regional government- judicial	+	Regional agency or department responsible for the legal system (e.g., courts)	Domestic Relations Court
	Regional government- justice/corrections	Regional agency or department responsible for administration of justice system (e.g., prison, law enforcement)	[county] police
Regional government- Medicaid/health	+		[county] public health department
	Regional government- disabilities	Similar substantive focus or purview to state government- disabilities, but smaller jurisdiction	Southwest region- Department of Disabilities
	Regional government- mental health	Similar substantive focus or purview to state government- mental health, but smaller jurisdiction	[county] Department of Mental Health
Regional government- other	—	Any regional department or agency not reflected in more specific codes or that crosses several codes	[county] Housing and Human Services
	Regional government- education		School Board
	Regional government	Equivalent to state legislature; related to governing body of elected or appointed officials responsible for a regional/local constituency	County Commissioner
	Regional board		
Non-governmental services			
Non-governmental, local service delivery	—	Organizations (for-profit or non-profit) and agencies responsible for delivering services such as health care	Crossnore School; Catholic Community Services; Independence Rising; Building Blocks Foster Care; Justice Works Behavioral Health Services
Special interests/experts*			
Non-governmental, Local advocacy	—	An association of individuals or formal organization (e.g., non-profit) with an advocacy-driven mission. Includes local	[state] Foster Youth Connection; Grandparent Kinship Coalition; BeNChmarks NC

		chapters of advocacy networks that have presence at higher levels (e.g., national)	
Non-governmental, Local service delivery and advocacy	—	Specifically mentions advocacy in addition to delivered programs or services, not just education about services	Lutheran Homes Society; Boys Town
	Alliance	Group of individuals united by a shared personal or professional identity. May or may not engage in advocacy or service delivery. If alliance or association in the name, coded as such; could be cross-level or state-level, as sometimes the state is in the organization's name	[state] Children's Services Alliance; [state] Foster Parent Association; Director's Association
**Tribal**			
Tribal government-primary	—	Primary tribal government entity, equivalent to state legislature or executive office	Confederated Tribes of Grand Ronde
Tribal government-child welfare	—	Agency or department tasked with child welfare and family services for a specific Tribe	Director of Tribal Social Services
Tribal government-other	—	Provides services as part of an arm of the tribal government	Human Services
Non-governmental Services			
Tribal services	—	Services specific to tribal Nations and communities; not run by tribal government; sometimes not affiliated with a unique department, but their role indicates a unique set of responsibilities with primary government entity	Indian Child Welfare Coalition
Special interests/experts*			
Tribal services and advocacy	—	Provides both services to tribe members and engages in advocacy	—
Tribal advocacy	—	Engages in advocacy for a specific tribe or alliance of Tribes	—
**Cross-level**			
Research/academic	—	Public or private institute of education or independent research organization (profit or non-profit)	University of [state]
Lived experience	—	Unaffiliated person with lived experience; coded at individual and organizational level	Youth Member
Managed Care Organization or other health insurance provider	—	Insurer, manage care organization, or community care organization responsible for members at varying levels	Trillium Family Services; Wellcare
	Professional association	Similar to *Alliance (below)*, but specifically marked by professional identity as individuals, not necessarily as a whole organization	Behavioral Health Services Association

	Alliance	Group of individuals united by a shared personal or professional identity. May or may not engage in advocacy or service delivery. If alliance or association in the name, coded as such; could be cross-level or state-level, as sometimes the state is in the organization's name	Primary Health Care Association
Missing	—	Insufficient information to determine organization affiliation, organization purpose, or distinguish between codes	—
**Individual Roles (Step 4)**			
Bureaucratic/political*			
Legislator		Elected member of state legislature	Representative
Elected official-other	—	Elected official that is responsible for local government services at a broad level or specific local/regional services	Sheriff; County or city Commissioner; Alder; City Board Member
Judge	—	Elected or appointed judge at any government level	Judge
Legislative Liaison	+	Policy administrator that is in a career track (i.e., not appointed or elected)	Legislative liaison; Legislative Aide
	Policy position within legislature	Policy advisor, researcher, administrator that is in a career track (i.e., not appointed or elected) yet employed by the state legislature or communicates between legislature and other state entities	Human Services Policy Advisor (in Office of the Governor)
Organization-specific decision influence			
Executive Leadership	*Combined with leadership*	*Not applicable; combined with leadership*	—
Leadership		Individual in highest or second-highest position of the organization/agency or any department/smaller unit within	Vice President; Director; Board of Director; Deputy Director; Executive Director; Deputy Secretary
Organization-specific services			
Middle Manager	Middle manager/ Supervisor	Supervisor or team leader responsible for supervision and guidance of front-line workers (e.g., clinicians) and potentially client care; Some administrative duties and responsibilities; Not specific to a particular program or policy effort; Responsible for developing and implementing little p policy	Child, Adolescent and Family Behavioral Health Services Manager; Social Services Manager; Foster care Systems Manager; Associate Director; Client Service Coordinator; Section Chief
	Program Manager	Administrative oversight and organizing activities specific to a particular program or policy effort	Foster Care Systems Manager; Child Fatality Prevention System Manager; Strengthening Families Director; Program Specialist; Program Specialist
	Contracting/ procurement	Any entities involved in developing, enforcing, and complying with contracts related to intervention delivery	Development Specialist; Contract Monitoring

Lived Experience	—	Individual operating in the conversation due to their lived experience relative to the policy at hand	Kinship Parent; Resource Parent; Foster Youth; Guardian ad litem
Administrator	—	Provides administrative assistance support	Legislative Administrator; Assistant Secretary for County Operations
Researcher/Analyst	—	Provides professional program evaluation or research evaluation; Can serve in advisory capacity, internal, or external/independent evaluation capacity	Strategic Integrated Policy Advisor; Performance Analyst
Practitioner	Practitioner-programmatic	Similar to middle managers, responsible for developing and implementing little p policies, but for more specific purposes (such as a specific program or substantively-focused set of programs); Unlike middle managers, these practitioners likely have more direct involvement with the staff responsible for service delivery (i.e., practitioner-direct client care)	Child Welfare Program Administrator; Child Fatality Prevention System Manager
	Practitioner- direct client care	Responsible for actively delivering therapeutic services	Society of Care Navigator; Family Engagement Director; Clinical Services Staff
Government Liaison	+	Liaison with government that operates within a non-governmental organization OR that operates in government organization to communicate with public	Government Liaison
	Policy advising within organization	Policy researcher or analyst for the purposes of improving organization-specific services or policies;	Senior Director for Policy and Planning; Senior Policy Advisor; Policy Administrator II
Special interests/experts*			
Advocate	—	Speaks out for/advocates for special interests of marginalized or underrepresented groups; may or may not have lived experience, but operate within a formal organization with an advocacy-driven mission	Youth member
Lived Experience	—	In the conversation/policy implementation process due to their lived experience relative to the policy at hand	Former foster youth
Legal Expert	—	A lawyer or scholar with legal expertise designated in their scope of work; Typically, this code only applied to individuals other than lawyers if explicitly described as such	Senior Juvenile Law Analyst
	Connector		Relationship Facilitator; Foster Care Liaison
Missing	—	Insufficient information to determine primary role or distinguish between codes	—

* from Bullock et al (2021) taxonomy;
\+ *Code maintained, definition changed;*
— no changes

Note: Some examples might be listed in multiple categories. This is intentional to indicate that the name alone cannot determine the code, but further investigation is required to understand the function of that individual or organization.
