# Supplementary material for: Who’s “in the room where it happens”? A taxonomy and five-step methodology for identifying and characterizing policy actors
Source: Implement Sci Commun. 2023 Sep 18;4:113. doi: 10.1186/s43058-023-00492-6 (PMC10506261; doi:10.1186/s43058-023-00492-6)
Supplement: Supplementary file 3 — Additional file 3. Suggested Reporting When Applying Five-Step Methodology for Identifying the Policy “Room” and Actors Within It. [file 43058_2023_492_MOESM3_ESM.pdf]

Additional File 3: Suggested Reporting When Applying Five-Step Methodology for Identifying the Policy “Room” and Actors Within It

| | **Question** | **Example Response or Documentation** |
|---|---|---|
| **1** | How was the level of code specificity determined? How were codes clearly distinguished from each other (definitions)? | A specific theory, framework, or method (e.g., literature review) that was used to create *a priori* codes. Detailed description of how emergent codes were identified and how the final set of codes was reached, including which codes were identified during co-coding. |
| **2** | How and where was identifying information gathered? | Google Search; LinkedIn; Conference Roster; Meeting minutes |
| **3** | How was co-coding conducted? | One coder developed and applied an initial codebook. Second coder reviewed codes for clarity and inquired about coding consistency, decision making, and sources of information. Iterative codebook discussed by two coders over three meetings. Final codebook applied by initial coder. |
| **4** | Why were certain policy actors designated as high priority (i.e., assigned an *) | High-priority actors were defined as individuals who would have final decision-making authority over what resources would be allocated to the policy implementation. |
